# A short-term high-fat diet alters rat testicular activity and blood-testis barrier integrity through the SIRT1/NRF2/MAPKs signaling pathways

**DOI:** 10.3389/fendo.2023.1274035

**Published:** 2023-10-27

**Authors:** Sara Falvo, Sergio Minucci, Alessandra Santillo, Rosalba Senese, Gabriella Chieffi Baccari, Massimo Venditti

**Affiliations:** ^1^ Dipartimento di Scienze e Tecnologie Ambientali, Biologiche e Farmaceutiche, Università degli Studi della Campania ‘Luigi Vanvitelli’, Caserta, Italy; ^2^ Dipartimento di Medicina Sperimentale, Sez. Fisiologia Umana e Funzioni Biologiche Integrate, Università degli Studi della Campania ‘Luigi Vanvitelli’, Napoli, Italy

**Keywords:** overweight, steroidogenesis, spermatogenesis, meiosis, mitochondria, inflammation

## Abstract

**Background:**

Overweight/obesity are metabolic disorder resulting from behavioral, environmental, and heritable causes. WHO estimates that 50% of adults and 30% of children and adolescents are overweight or obese, and, in parallel, an ongoing decline in sperm quality and male fertility has been described. Numerous studies demonstrated the intimate association between overweight/obesity and reproductive dysfunction due to a highly intricate network of causes not yet completely understood. This study expands the knowledge on the impact of a short-term high-fat diet (st-HFD) on rat testicular activity, specifically on steroidogenesis and spermatogenesis, focusing on the involved molecular mechanisms related to mitochondrial dynamics, blood-testis barrier (BTB) integrity, and SIRT1/NRF2/MAPKs pathways.

**Methods:**

Ten adult Male Wistar rats were divided into two groups of five and treated with a standard diet or an HFD for five weeks. At the end of the treatment, rats were anesthetized and sacrificed by decapitation. Blood was collected for serum sex hormone assay; one testis was stored at -80ÅãC for western blot analysis, and the other, was fixed for histological and immunofluorescence analysis.

**Results:**

Five weeks of HFD results in reduced steroidogenesis, increased apoptosis of spermatogenic cells, and altered spermatogenesis, as highlighted by reduced protein levels ofmeiotic and post-meiotic markers. Further, we evidenced the compromission of the BTB integrity, as revealed by the downregulation of structural proteins (N-Cadherin, ZO-1, occludin, connexin 43, and VANGL2) other than the phosphorylation of regulative kinases (Src and FAK). At the molecular level, the impairment of mitochondrial dynamics (fission, fusion, andbiogenesis), and the dysregulation of the SIRT1/NRF2/MAPKs signaling pathways, were evidenced. Interestingly, no change was observed in the levels of pro-inflammatory markers (TNFα, NF-kB, and IL-6).

**Conclusions:**

The combined data led us to confirm that overweight is a less severe state than obesity. Furthermore, understanding the molecular mechanisms behind the association between metabolic disorders and male fertility could improve the possibility of identifying novel targets to prevent and treat fertility disorders related to overweight/obesity.

## 1 Introduction

An intimate connection between balanced nutrition and the preservation of a good state of human health exists, in fact a salubrious diet is associated with a reduction in morbidity and premature mortality (1–3). Many studies reported that, especially in industrialized countries, a considerable percentage of non-communicable diseases (obesity, diabetes, cardiovascular disorders, and even some types of cancer) are correlated, directly or indirectly, to the consumption of unhealthy food, particularly those with the high trans-fatty acids and low essential nutrients content (vitamins, minerals, and proteins) (4–6). It has been estimated that obesity and overweight, syndromes characterized by the accumulation of excessive fatty tissue in the body, affect more than 1.9 billion adults worldwide, rising from epidemic to pandemic states (7). Such high prevalence, accompanied by severe social and economic consequences, makes obesity/overweight one of the major global health issues (8). It is important to note that being overweight may be considered a preclinical condition less severe than obesity, since the excessive accumulation of body fat increases, in turn, the risk of chronic diseases (9). The most used parameter to define obesity is the body mass index (BMI), calculated as a person’s weight (in kilograms) divided by the square of his/her height (in meters) (10). Conversely, more accurate but less used indexes, such as waist circumference and weight gain, may provide more reliable and individualized parameters to define the consequence of excessive body fat accumulation on the development of chronic disease (11). Obesity rates have significant impacts on personal and public health; however, overweight status is often trivialized as a mere body image issue (12, 13).

Besides the well-known comorbidities associated with obesity, including dyslipidemia, type 2 diabetes, and hypertension, a growing body of evidence is now focusing on its correlation with human infertility, as evidenced by the numerous papers published on this topic in recent years and, in particular, on the positive correlation between growing BMI and sub-infertility (14–16). Alteration of the hormonal milieu is one of the most evident effects of obesity. In overweight or obese men, excess body fat accumulation can increase the production of serum sex hormone-binding globulin. This glycoprotein, produced by the liver, binds to testosterone (T) and inhibits its biological action; this, along with increased aromatase (ARO) activity, leads to a decreased T/estradiol (E_2_) ratio; estrogen increases and, inhibiting Leydig and Sertoli cell function, further impairs T production and the process of spermatogenesis (17–20).

Moreover, obesity has also been defined as a “systemic oxidative stress state”, in which an imbalance between reactive oxygen species (ROS) production and antioxidant capacity occurs, leading to oxidative stress. This, ultimately, damages cellular components deleterious for male germ cells (GC), and particularly for spermatozoa (SPZ), as their plasma membrane contains high levels of polyunsaturated fatty acids, and their DNA, once damaged, cannot be repaired due to lack of the cytoplasmic enzymatic systems involved in DNA repair (17, 21–23). Several studies reported that, compared to normal-weight men, obese ones have a higher chance of oligozoospermia, asthenozoospermia, and an increased rate of fragmented DNA in sperm (24–28). Furthermore, in a meta-analysis, Campbell et al. (29) described that male obesity negatively impacts the success of assisted reproductive technology (ART). Interestingly, while changes in sex hormone levels may contribute to obesity-induced male sub-infertility, data from ART indicate that they may not be the only cause; in fact, obesity in men is associated with decreased pregnancy rates and increased pregnancy loss in couples subjected to ART, but, following intracytoplasmic sperm injection, the fertilization rate is considerably improved, indicating that obesity may alter sperm maturation, capacitation, and their ability to bind and fertilize the egg with still unknown mechanisms (29–31). In this regard, one of the most common tools to study obesity and its related comorbidities, including infertility, is the use of animal models, especially mice and rats, fed with a high-fat diet (HFD). The duration of the HFD is crucial; in a recent review, de Moura e Dias et al. (32) summarized the time-dependent effects of HFD in provoking obesity, assessing that at least 3 weeks of HFD are sufficient to obtain satisfactory results. However, to strengthen the phenotypic and metabolic characteristics of obesity, a longer intervention period (from 10 to 12 weeks) is necessary. Coherently, most of the studies focused on the impact of obesity on testicular activity, used a long-term HFD (10-14 or longer weeks of treatment) (33–36), while just a few papers used a different approach, with a short-term HFD (st-HFD), that is correlated to an overweight condition (37–39).

This may be interesting to obtain parameters to be used to monitor the progression of infertility related to being overweight, even at the early stages before it progresses to obesity, which is considered a real “pathological state”. In previous studies, we demonstrated that a 5-weeks st-HFD induced an increase in body weight and serum cholesterol and triglyceride levels, as well as alterations in testis and epididymis, i.e., induced oxidative stress, increased autophagy, apoptosis, and mitochondrial damage (40–42). Here, using the same rats fed with a st-HFD, we evaluated additional parameters of testicular activity, such as steroidogenesis and spermatogenesis, with special attention to the involved mechanisms related to mitochondrial dynamics, and blood-testis barrier (BTB) integrity. Undoubtedly, these key regulators are essential in the spermatogenic process, which guarantees the formation of high-quality gametes (43, 44); on the other hand, testicular cells mitochondria and BTB are two of the main targets highly sensitive to the non-physiological conditions, and particularly in a prooxidant milieu, induced either by environmental (such as the exposure to pollutants) (45–49), and pathological (like diabetes and obesity) (50–52) factors. Finally, because many reports demonstrated the association of SIRT1/NRF2/MAPKs pathways with testicular function altered by obesity (33, 53–55), we verified whether the abovementioned pathways may also be involved in the molecular mechanisms underlying the diet-induced testicular dysfunction obtained via a st-HFD.

## 2 Methods

### 2.1 Animals and tissue collection

Male Wistar rats (250–300 g, aged eight weeks) were kept in one per cage in a temperature-controlled room at 28°C (thermoneutrality for rats) under a 12-h light/12-h dark cycle. Before the beginning of the study, water, and a commercial mash (Charles River Laboratories, Calco, Italy) were available *ad libitum*. At the start of the study (day 0), and after seven days of acclimatization to thermoneutrality, the rats were divided into two groups of five and treated as follows:

- The first group of rats (n = 5, C) received a standard diet (total metabolizable percentage of energy: 60.4 carbohydrates, 29 proteins, 10.6 fat J/J; 15.88 kJ gross energy/g; Muscedola, Milan, Italy) for five weeks;
- The second group of rats (n = 5, st-HFD) received a HFD (280 g diet supplemented with 395 g of lyophilized lamb meat (Liomellin, Milan, Italy), 120 g cellulose (Sigma-Aldrich, St. Louis, MO, USA), 20 g mineral mix (ICN Biomedical, Solon, OH, USA), 7 g vitamin mix (ICN), and 200 g low-salt butter (Lurpak, Denmark). Approximate fatty acid profile of this diet was: 45% saturated (SFA), 45% MUFA, 10% PUFA. total metabolizable percentage of energy: 21 carbohydrates, 29 proteins, 50 fat J/J; 19.85 kJ gross energy/g) for five weeks.

At the end of the treatment, rats were anesthetized with intraperitoneal injection of chloral hydrate (40mg/100g body weight), sacrificed for decapitation. The trunk blood was collected and the serum was separated and stored at -20°C for later sex hormone determination. The testes were dissected out, one testis was rapidly immersed in liquid nitrogen and stored at -80°C for western blot (WB) analysis, and the other was fixed in Bouin’ solution for histological analysis. This study is reported in accordance with ARRIVE guidelines. Animal care and experiments were conducted in accord with the guidelines of the Ethics Committee of the University of Campania “Luigi Vanvitelli” and the Italian Minister of Health (Permit Number: 704/2016-PR of the 15/07/2016; Project Number: 83700.1 of the 03/05/2015). Every effort was made to minimize animal pain and suffering.

### 2.2 Determination of serum T and E_2_ levels

Sex steroid levels were determined in serum from control and st-HFD rats using T (#DKO002; DiaMetra, Milan, Italy) and E_2_ (#DKO003; DiaMetra, Milan, Italy) enzyme immunoassay kits. The sensitivities were 32 pg/mL for T and 15 pg/mL for E_2_.

### 2.3 Protein extraction and WB analysis

Total testicular proteins were extracted from control (n = 5) and st-HFD (n = 5) rats as described in Venditti et al. (56). Forty micrograms of total protein extracts were separated into SDS-PAGE (9 or 15% polyacrylamide) and treated as described in Venditti et al. (57). The membranes were incubated overnight at 4°C with primary antibodies, listed in **Table S1**. The concentration of proteins was quantified using ImageJ software (version 1.53 t; National Institutes of Health, Bethesda, USA). Each WB was performed in triplicate.

### 2.4 Histology and immunofliorescence (IF) analysis

For hematoxylin/eosin staining and immunolocalization analysis, 5 µm testis sections were dewaxed, rehydrated, and processed as previously described (58, 59). For details on the used antibodies, see **Table S1**. The cells’ nuclei were marked with Vectashield + DAPI (Vector Laboratories, Peterborought, UK) and then observed under an optical microscope (Leica DM 5000 B + CTR 5000; Leica Microsystems, Wetzlar, Germany) with UV lamp, images were analyzed and saved with IM 1000 software (version 4.7.0; Leica Microsystems, Wetzlar, Germany). Photographs were taken using the Leica DFC320 R2 digital camera. Densitometric analysis of IF signals and Proliferating Cell Nuclear Antigen (PCNA)/Synaptonemal complex protein 3 (SYCP3) positive cells were performed with Fiji plugin (version 3.9.0/1.53 t) of ImageJ Software counting 30 seminiferous tubules/animal for a total of 150 tubules per group. Each IF was performed in triplicate.

### 2.5 TUNEL assay

The apoptotic cells were identified in paraffin sections through the DeadEnd™ Fluorometric TUNEL System (#G3250; Promega Corp., Madison, WI, USA) following the manufacturer’s protocol, with little modifications. Briefly, before the incubation with TdT enzyme and nucleotide mix, sections were blocked with 5% BSA and normal goat serum diluted 1:5 in PBS and then treated with PNA lectin, to mark the acrosome. Finally, the nuclei of the cells were counterstained with Vectashield + DAPI. The sections were observed with the same microscope described in Section 2.4. To determine the % of TUNEL-positive cells, 30 seminiferous tubules/animal for a total of 150 tubules per group, were counted using the Fiji plugin (version 3.9.0/1.53 t) of ImageJ Software. TUNEL assay was performed in triplicate.

### 2.6 Statistical analysis

The values were compared by a Student’s t-test for between-group comparisons using Prism 8.0, GraphPad Software (San Diego, CA, United States). Values for p < 0.05 were considered statistically significant. All data were expressed as the mean ± standard error mean (SEM).

## 3 Results

### 3.1 Effect of st-HFD on testicular steroidogenesis

**Figure 1:**
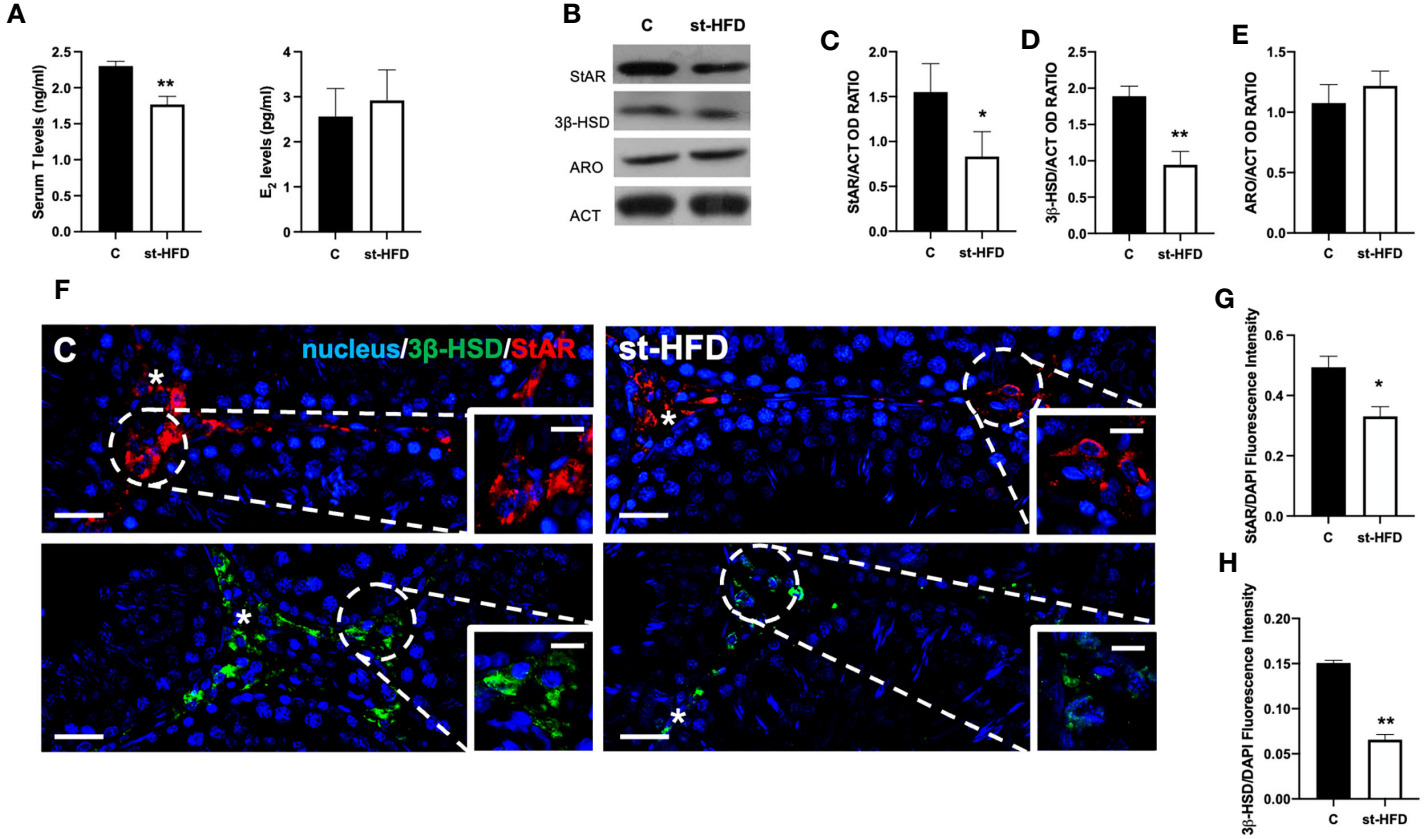
Steroidogenesis analysis of controls and st-HFD fed rat testis. **(A)** T and E_2_ serum levels and **(B)** WB analysis of testicular StAR, 3β-HSD and ARO protein levels. **(C-E)** Histograms showing StAR, 3β-HSD, and ARO relative protein levels. **(F)** Testicular StAR (red) and 3β-HSD (green) immunolocalization. Slides were counterstained with DAPI-fluorescent nuclear staining (blue). The images were captured at x20 (scale bars= 20 µm) magnification and x40 (scale bars= 10 µm) for the insets. Asterisks: LC. **(G, H)** Histogram showing the quantification of StAR and 3β-HSD fluorescence signal intensity, respectively. All values are expressed as means ± SEM from 5 animals in each group. *p < 0.05; **p < 0.01.

Serum T levels in st-HFD rats were significantly reduced by about 28% compared to the controls (p < 0.01); by contrast no differences in E_2_ levels between the two groups were evidenced (**Figure 1A**).

To better evaluate the effect of st-HFD on steroidogenesis, the protein levels of steroidogenic acute regulatory protein (StAR), and 3β-Hydroxysteroid dehydrogenase (3β-HSD), two enzymes involved in T biosynthesis, were analyzed (**Figure 1B**). WB analysis confirmed that st-HFD altered testicular steroidogenesis, as a decrease in StAR (p < 0.05; **Figures 1B, C**) and 3β-HSD (p < 0.01; **Figures 1B, D**) protein levels, as compared to the control, was observed. In addition, the protein level of ARO, the enzyme converting T into E_2_, was also evaluated, however, results showed no difference between the two groups (**Figures 1B, E**)

The effects of st-HFD on steroidogenesis were further confirmed by an IF staining of StAR and 3β-HSD, which is shown in **Figure 1F**. The signals specifically localized into the interstitial Leydig cells (LC; asterisks; **Figure 1F** insets); however, fluorescence intensity analysis showed a weaker signal in st-HFD animals (p < 0.01; **Figures 1G, H**) as compared to the control.

### 3.2 Effect of st-HFD on apoptosis

**Figure 2:**
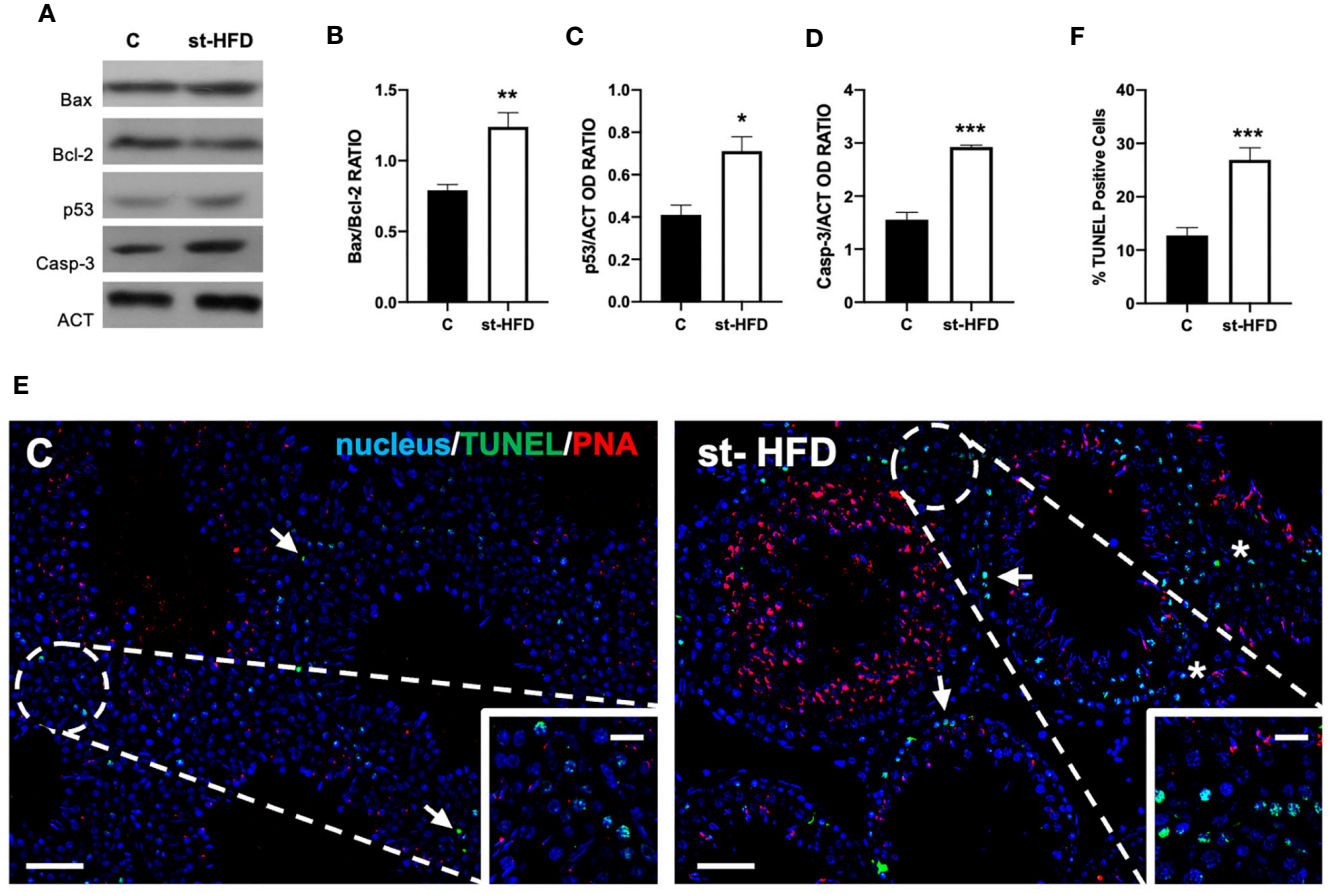
Apoptosis rate analysis of control and st-HFD fed rat testis. **(A)** WB analysis of testicular Bax, Bcl-2, p53, and Caspase-3. **(B-D)** Histograms showing the Bax/Bcl-2 ratio, p53, and Caspase-3 relative protein levels. **(E)** Determination of apoptotic cells through the detection of TUNEL-positive cells (green). Slides were counterstained with PNA lectin (red) and with DAPI-fluorescent nuclear staining (blue). The images were captured at x10 magnification (scale bars= 20 µm) and x20 (scale bars= 10 µm) for the insets. Arrows: SPG; Asterisks: LC. **(F)** Histogram showing the % of TUNEL-positive cells. All the values are expressed as means ± SEM from 5 animals in each group. *p < 0.05; **p < 0.01; ***p < 0.001.

**Figure 2** shows the effect of st-HFD on the apoptotic rate of germ and somatic cells. WB analysis revealed an increase in Bax/Bcl-2 ratio (p < 0.01; **Figures 2A, B**), p53 (p < 0.05; **Figures 2A, C**), and Caspase-3 (p < 0.001; **Figures 2A, D**) protein levels in the st-HFD group as compared to the control.

In support of these data, a TUNEL assay was performed (**Figure 2E**). Data showed the presence of dispersed apoptotic cells in the control group, especially spermatogonia (SPG; arrows and insets; **Figure 2E**). st-HFD induced an increase of 165% in the number of TUNEL-positive cells (p < 0.001; **Figures 2E, F**), particularly of SPG, as well as scattered apoptotic LC in the interstitial compartment, as related to the control.

### 3.3 Effect of st-HFD on spermatogenesis

**Figure 3:**
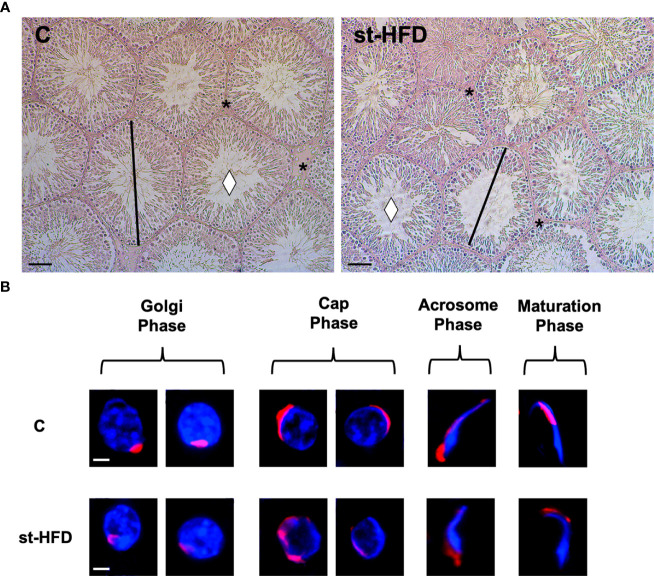
Histological analysis of control and st.HFD fed rat testis. **(A)** Hematoxylin-eosin staining of rat testicular paraffin-embedded sections. The images were captured at x20 magnification (scale bars= 40 µm). Rhombus: tubules lumen; asterisks: interstitial compartment. **(B)** PNA lectin acrosome staining (red) showing the different phases of acrosome biogenesis. The images were captured at x40 magnification (scale bars= 10 µm).

Testis from control exhibited well-organized germinal and interstitial compartment, showing GC in all differentiation stages and with mature SPZ filling tubular lumina (rhombus) as well as LC and regular blood vessels in the interstitium (asterisk; **Figure 3A**). The histological organization of the testes from st-HFD rats was not dissimilar from that of controls; however, it appeared clear the reduced diameter of the tubules. Indeed, the analysis of three morphometric parameters further supported this observation since the diameter of the tubules (p < 0.001) and the thickness of epithelium (p < 0.05) were lower in st-HFD group than in the control, while no differences in the % of tubular lumens occupied by SPZ were detected (**Table 1**). In addition, although there were no changes in the frequency of the stages characterizing the rat seminiferous epithelium (data not shown), alterations in the different phases of the acrosome biogenesis, highlighted by the PNA lectin staining, were seen (**Figure 3B**).

**Table 1:** Effect of st-HFD on testicular morphometric parameters.

Groups	C	st-HFD
Tubules Diameter (µm)	225,32± 2,17	171,49 ± 6,38^**^
Epithelium Thickness (µm)	43,2 ± 1,13	30,51 ± 2,4^*^
Empty Lumen (%)	36 ± 2,3	39 ± 1,5

Evaluation of testicular morphometric parameters of control and st-HFD fed rat testis. All the values are expressed as means ± SEM from 5 animals in each group. *p < 0.05; **p < 0.01.

**Figure 4:**
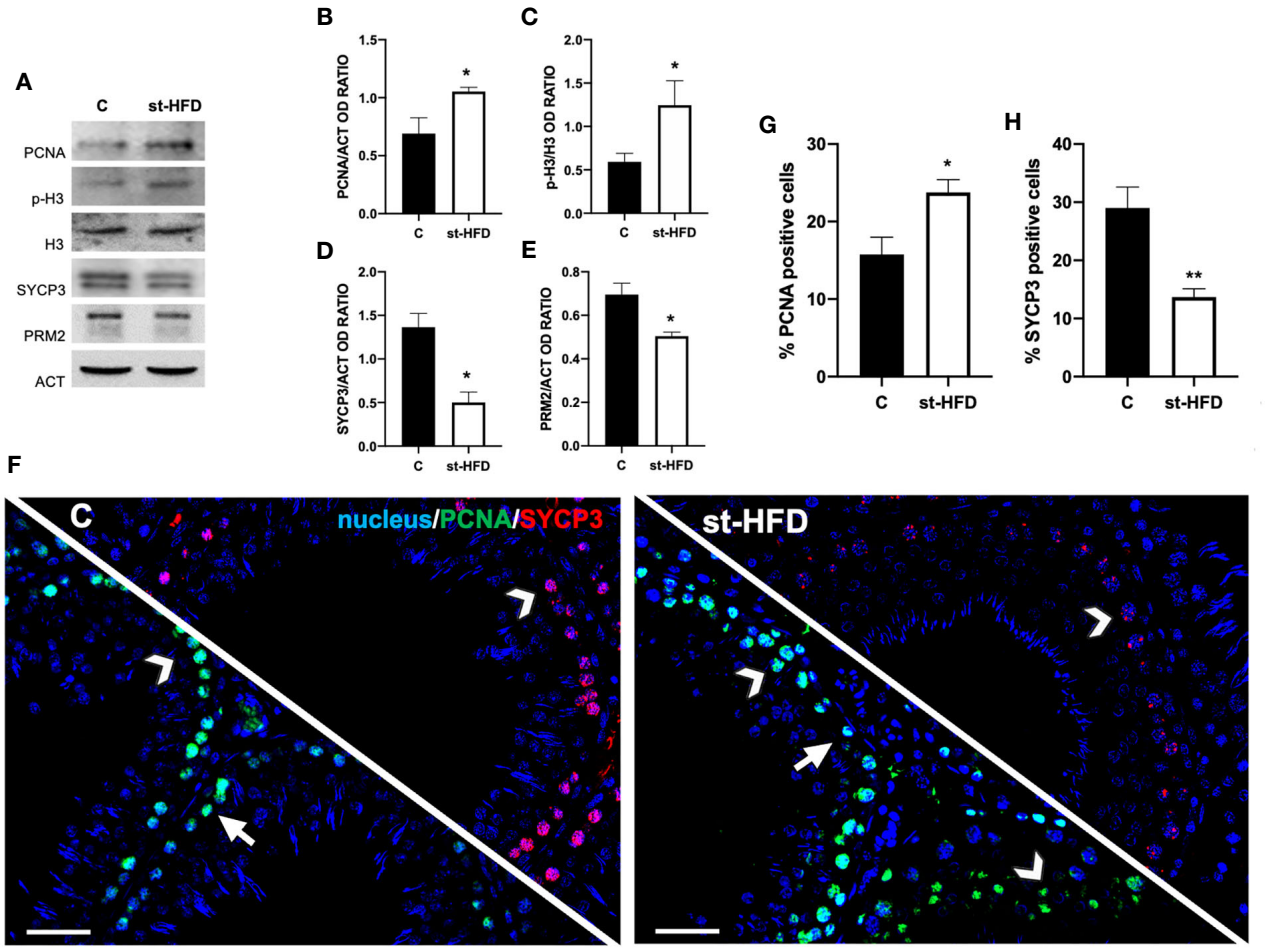
Spermatogenesis analysis of control and st-HFD fed rat testis. **(A)** WB analysis of testicular PCNA, p-H3, H3, SYCP3, and PRM2. **(B-E)** Histograms showing the p-H3/H3 ratio, PCNA, SYCP3, and PRM2 relative protein levels. **(F)** Testicular PCNA (green) and SYCP3 (red) immunolocalization. Slides were counterstained with DAPI-fluorescent nuclear staining (blue). The images were captured at x20 magnification (scale bars=20 µm). Arrows: SPG; Arrowheads: SPC. **(G, H)** Histograms showing the % of PCNA and SYCP3 positive cells, respectively. All the values are expressed as means ± SEM from 5 animals in each group. *p < 0.05; **p < 0.01.

At molecular level, to evaluate the effects of st-HFD on spermatogenesis, protein levels of PCNA, phospho-histone H3 (p-H3), SYCP3, and protamine 2 (PRM2) were investigated (**Figures 4A-E**). The st-HFD provoked a significant increase (p < 0.05) in PCNA (**Figures 4A, B**), and p-H3 (**Figures 4A, C**), and a decrease (p < 0.05) in SYCP3 (**Figures 4A, D**) and PRM2 (**Figures 4A, E**) protein levels as related to the controls.

Concomitantly, labeling of PCNA and SYCP3 was performed (**Figure 4F**). Data showed a PCNA (green panel) specific localization in the SPG (arrows) and spermatocytes (SPC; arrowheads) in the testis of both groups; however, in st-HFD an increase approximately of 51% in PCNA positive cells (p < 0.05; **Figure 4G**) was observed. As for SYCP3, it localized in the SPC nucleus (arrowheads; **Figure 4E**), and the % of SYCP3 positive cells decreased by 53% in st-HFD group as compared to the control (p < 0.01; **Figure 4H**).

### 3.4 Effect of st-HFD on biogenesis and mitochondria dynamics

**Figure 5:**
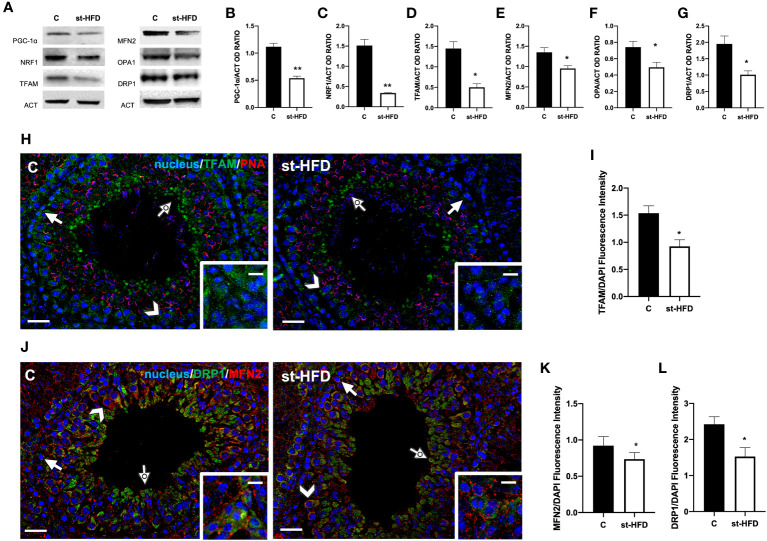
Mitochondrial dynamics analysis of control and st-HFD fed rat testis. **(A)** WB analysis of testicular PGC-1α, NRF1, TFAM, MFN2, OPA1, and DRP1. **(B-G)** Histograms showing PGC-1α, NRF1, TFAM, MFN2, OPA1, and DRP1 relative protein levels. **(H)** Testicular TFAM (green) immunolocalization. Slides were counterstained with PNA lectin (red). **(J)**. Testicular DRP1 (green) and MFN (red) immunolocalization. All the slides were counterstained with DAPI-fluorescent nuclear staining (blue). The images were captured at x20 (scale bars= 20 µm) magnification and x40 (scale bars= 10 µm) for the insets. Arrows: SPG; Arrowheads: SPC; Dotted arrows: SPT. Insets show LC. **(I, K, L)** Histograms showing the quantification of TFAM, MFN2, and DRP1 fluorescence signal intensity, respectively. All the values are expressed as means ± SEM from 5 animals in each group. *p < 0.05; **p < 0.01.

To evaluate the effects of st-HFD on mitochondrial biogenesis, peroxisome proliferator-activated receptor-gamma coactivator (PGC-1α), nuclear respiratory factor 1 (NRF1), and mitochondrial transcription factor A (TFAM) were employed as markers. We found a significant decrease in the expression levels of PGC-1α (p < 0.01; **Figures 5A, B**), NRF1 (p < 0.01; **Figures 5A, C**), and TFAM (p < 0.05; **Figures 5A, D**) in the testis of st-HFD rats as compared to controls.

Mitofusin (MFN2) and Optic atrophy 1 (OPA1) were employed as markers of mitochondrial fusion; Dynamin-Related Protein 1 (DRP1) was used as a marker of the fission process. Testes from st-HFD rats exhibited a slight, significant decrease in MFN2 (p < 0.05; **Figures 5A, E**), OPA1 (p < 0.05; **Figures 5A, F**), and DRP1 (p < 0.05; **Figure 5A, G**) protein levels as compared to control animals.

IF staining was performed for TFAM (**Figure 5H**), MFN2, and DRP1 (**Figure 5J**). In the control testis, TFAM localized in the cytoplasm of SPG (arrows), SPC (arrowhead), and in the residual cytoplasm of elongating spermatids (SPT; dotted arrows). Additionally, a clear signal in the interstitial LC was also observed (insets). In the st-HFD-treated group, TFAM localized in the same cell types abovementioned (**Figure 5H**), but a weaker immunofluorescent signal was observed (p < 0.05; **Figure 5I**). Similarly, DRP1 also localized in the cytoplasm of SPG (arrow), SPC (arrowheads), in eltongating SPT (dotted arrows), as well as in LC (insets); interestingly, MFN2 signal appeared dotted-shaped and diffused in all the cell types composing the seminiferous epithelium. The analysis of MFN2 (**Figure 5K**) and DRP1 (**Figure 5L**) fluorescent signals showed a comparable pattern, statistically significant, as observed for the protein level.

### 3.5 Effect of st-HFD on BTB integrity markers

**Figure 6:**
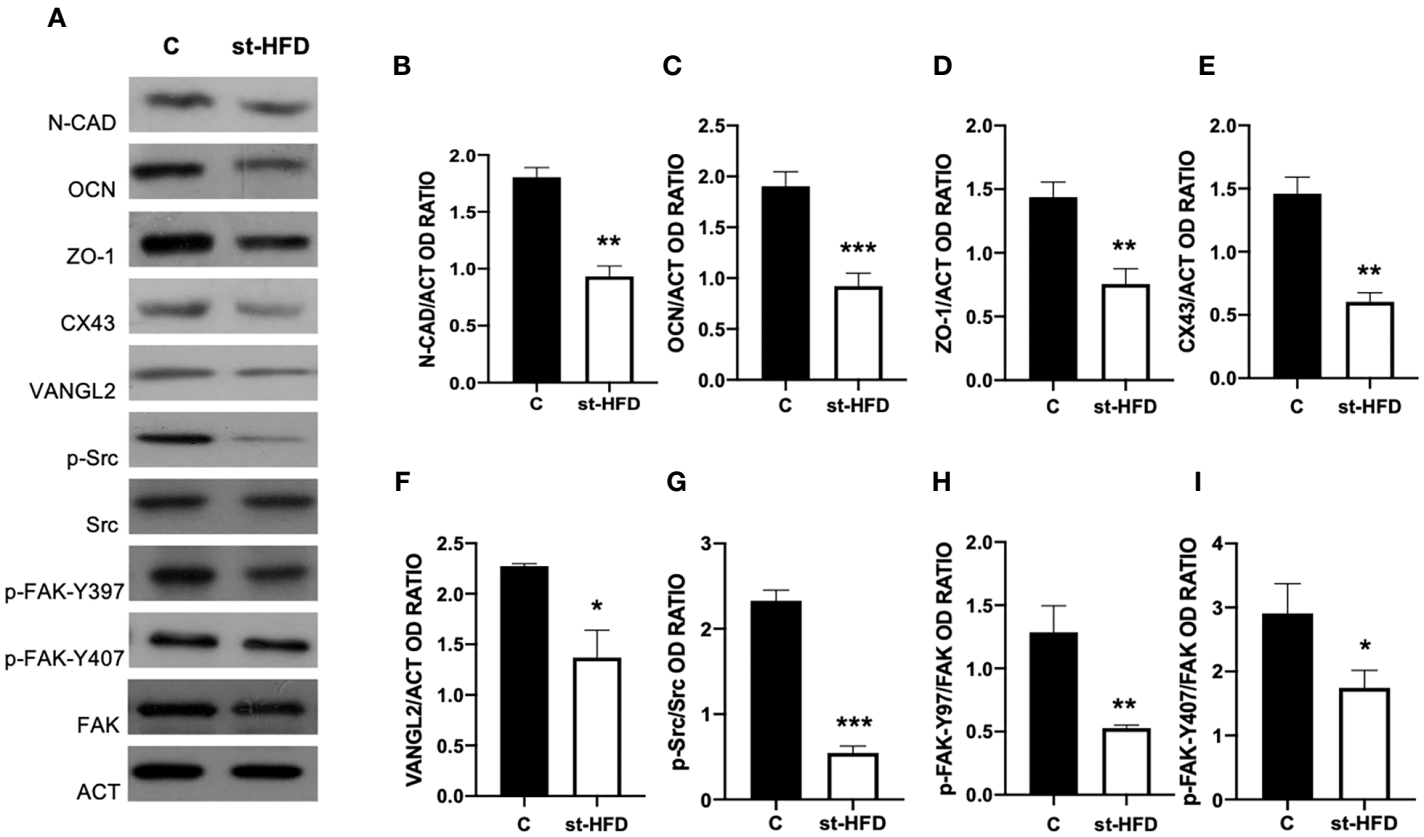
BTB markers analysis of control and st-HFD fed rat testis. **(A)** WB analysis of testicular N-CAD, OCN, ZO-1, CX43, VANGL2, p-Src, Src, p-FAK-Y397, p-FAK-Y407, and FAK, in the testes of animals treated with a st-HFD. **(B–I)** Histograms showing N-CAD, OCN, ZO-1, CX43, and VANGL2 relative protein levels, and p-Src/Src, p-FAK-Y39/FAK, and p-FAK-Y407/FAK ratios. All the values are expressed as means ± SEM from 5 animals in each group. *p < 0.05; **p < 0.01; ***p < 0.001.

**Figure 7:**
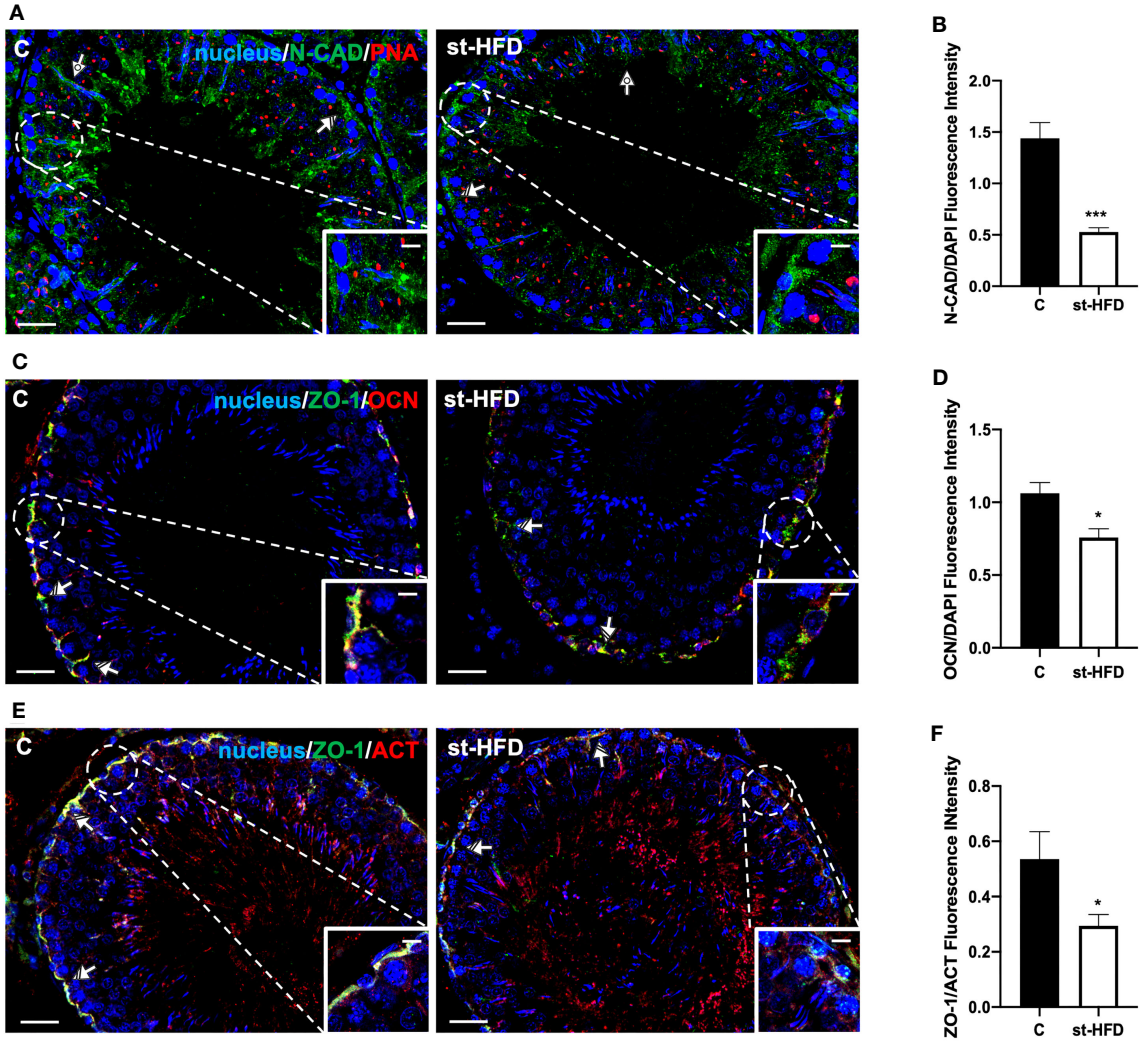
IF analysis of N-CAD, ZO-1, and OCN of control and st-HFD fed rat testis. **(A)** Testicular N-CAD (green) immunolocalization. Slides were counterstained with PNA lectin (red) and DAPI-fluorescent nuclear staining (blue). **(C)** Testicular ZO-1 (green) and OCN (red) immunolocalization. **(E)** Testicular ZO-1 (green) and β-Actin (red) immunolocalization. All the slides were counterstained with DAPI-fluorescent nuclear staining (blue). All the images were captured at x20 (scale bars= 20 µm) magnification and x40 (scale bars= 10 µm) for the insets. Striped arrows: SC. Dotted arrows: SPT. **(B, D, F)** Histograms showing the quantification of N-CAD, OCN, and ZO-1 fluorescence signal intensity, respectively. All the values are expressed as means ± SEM from 5 animals in each group. *p < 0.05; ***p < 0.001.

**Figure 8:**
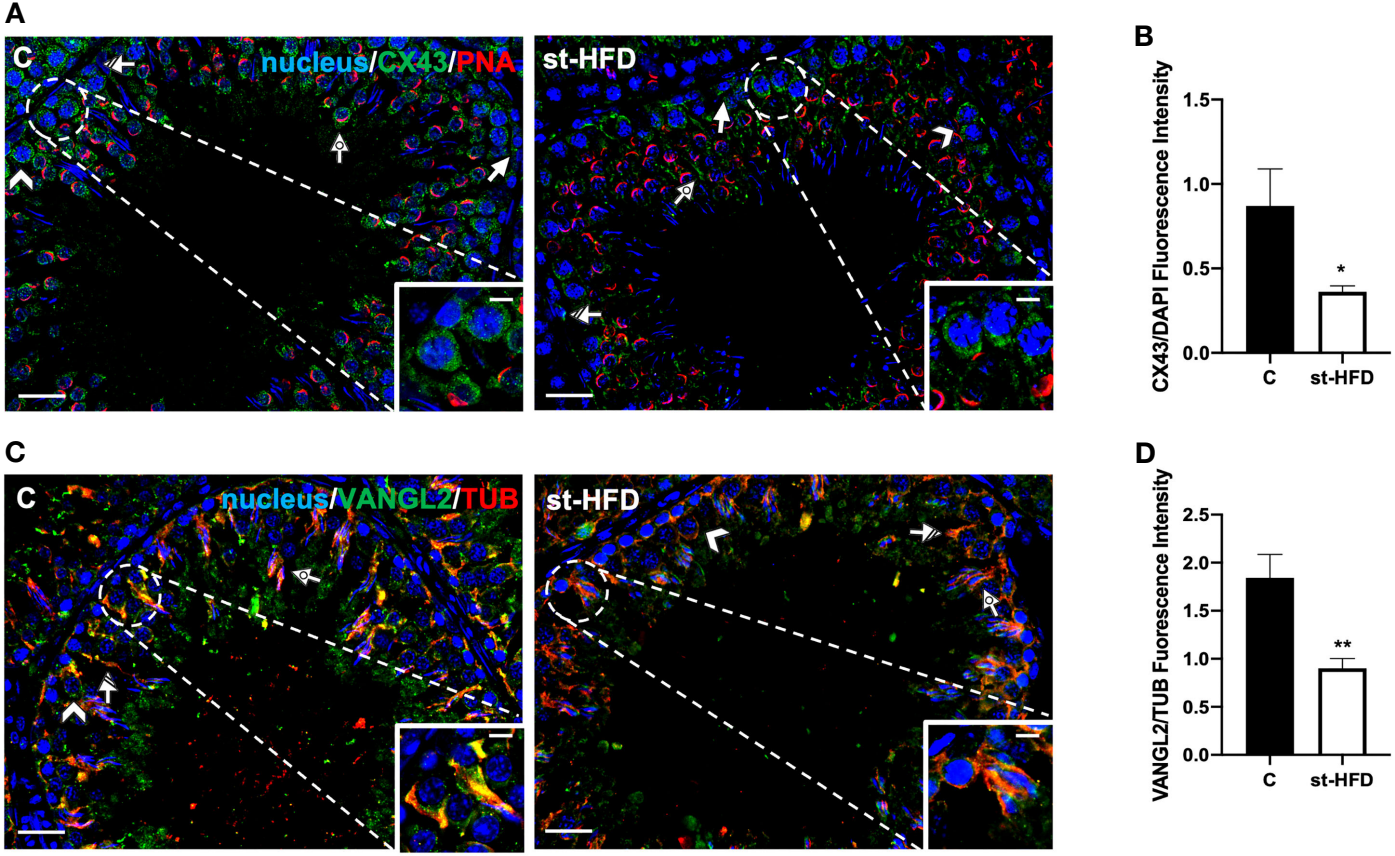
IF analysis of CX43, and VANGL2 of control and st-HFD fed rat testis. **(A)** Testicular CX43 (green) immunolocalization. **(C)** Testicular VANGL2 (green) and α-Tubulin (red) immunolocalization. All the slides were counterstained with DAPI-fluorescent nuclear staining (blue). All the images were captured at x20 (scale bars= 20 µm) magnification and x40 (scale bars= 10 µm) for the insets. Arrows: SPG; Arrowheads: SPC Dotted arrows: SPT. Striped arrows: SC. **(B, D)** Histograms showing the quantification of CX43 and VANGL2 fluorescence signal intensity, respectively. All the values are expressed as means ± SEM from 5 animals in each group. *p < 0.05; **p < 0.01.

st-HFD produced substantial alterations in the BTB at both structural and regulatory proteins, compared to control groups (**Figures 6**–**8**). Indeed, st-HFD resulted in a significant reduction in the protein levels of N-Cadherin (N-CAD; p < 0.01; **Figures 6A, B**), occludin (OCN; p < 0.001; **Figures 5A, C**), zonula occludens-1 (ZO-1; p < 0.01; **Figures 6A, D**), connexin 43 (CX43; p < 0.01; **Figures 6A, E**), and Van Gogh-Like 2 (VANGL2; p < 0.05; **Figure 6A, F**), as well as in the phosphorylation status of p-Src (p < 0.001; **Figures 6A, G**), p-FAK-Y397 (p < 0.01; **Figures 6A, H**), and p-FAK-Y407 (p < 0.05; **Figures 6A, I**) as compared to control.

For a more detailed characterization of the effects exerted by st-HFD on N-CAD, OCN, ZO-1 (**Figure 7**) CX43, and VANGL2 (**Figure 8**) localization, an IF analysis was carried out. N-CAD, one of the components of cell adhesion complexes (adhesion junctions) in BTB (60), localized both in the basal compartment, at Sertoli cells (SC) interface (striped arrows; **Figure 7A**), and in their cytoplasmic protrusions of the luminal compartment, associated with the heads of elongating SPT (dotted arrows; **Figure 6A**). Interestingly, in the testis of st-HFD-treated rats, while N-CAD immunosignal was still present in the basal compartment, in the luminal one it was quite weak, and less intense that of the control group (p < 0.001; **Figures 7A, B**).

OCN (**Figure 7C**) and ZO-1 (**Figure 7E**) are integral membrane and adaptor proteins, respectively, that link integral membrane tight junctions (TJ) components to the actin cytoskeleton (61). They specifically localized in the SC cytoplasm (striped arrows; **Figures 7C, E**; insets) in the two groups; however, the signal intensity decreased in the st-HFD-treated rats (p < 0.05; **Figures 7D, F**) as compared to the control.

CX43 is the principal testicular gap-junction protein, localized between adjacent SC and at the SC-GC interface (62). IF data confirmed this localization pattern; in control, CX43 was detected in the above-mentioned cell types, particularly in SPG (arrows; **Figure 8A**), SPC (arrowheads; **Figure 8A**; insets), SC (striped arrows; **Figure 8A**), and their cytoplasmic protrusions surrounding SPT (dotted arrows; **Figure 8A**). st-HFD produced a marked decrease of signal intensity in SC and GC, as compared to the control (p < 0.05; **Figure 8B**).

Finally, VANGL2 is a member of the Planar Cell Polarity family, factors that regulate the spatial and temporal expression of actin-regulatory proteins and the polymerization of microtubules at the apical ectoplasmic specialization (ES) and SC-SC and SC-SPT interface levels (63, 64). In the control testis, VANGL2 localized in SPC (arrowheads; **Figure 8C**), in the SC cytoplasm (striped arrows; **Figure 8C**; insets), and their protrusions surrounding the SPT/SPZ heads (dotted arrows; **Figure 8C**). In the st-HFD-treated group, although VANGL2 localized in the above-mentioned cell types (**Figure 8C**), a weaker immunofluorescent signal was observed (p < 0.01; **Figure 8D**).

### 3.6 Effect of st-HFD on SIRT1/NRF2/MAPKs pathways

**Figure 9:**
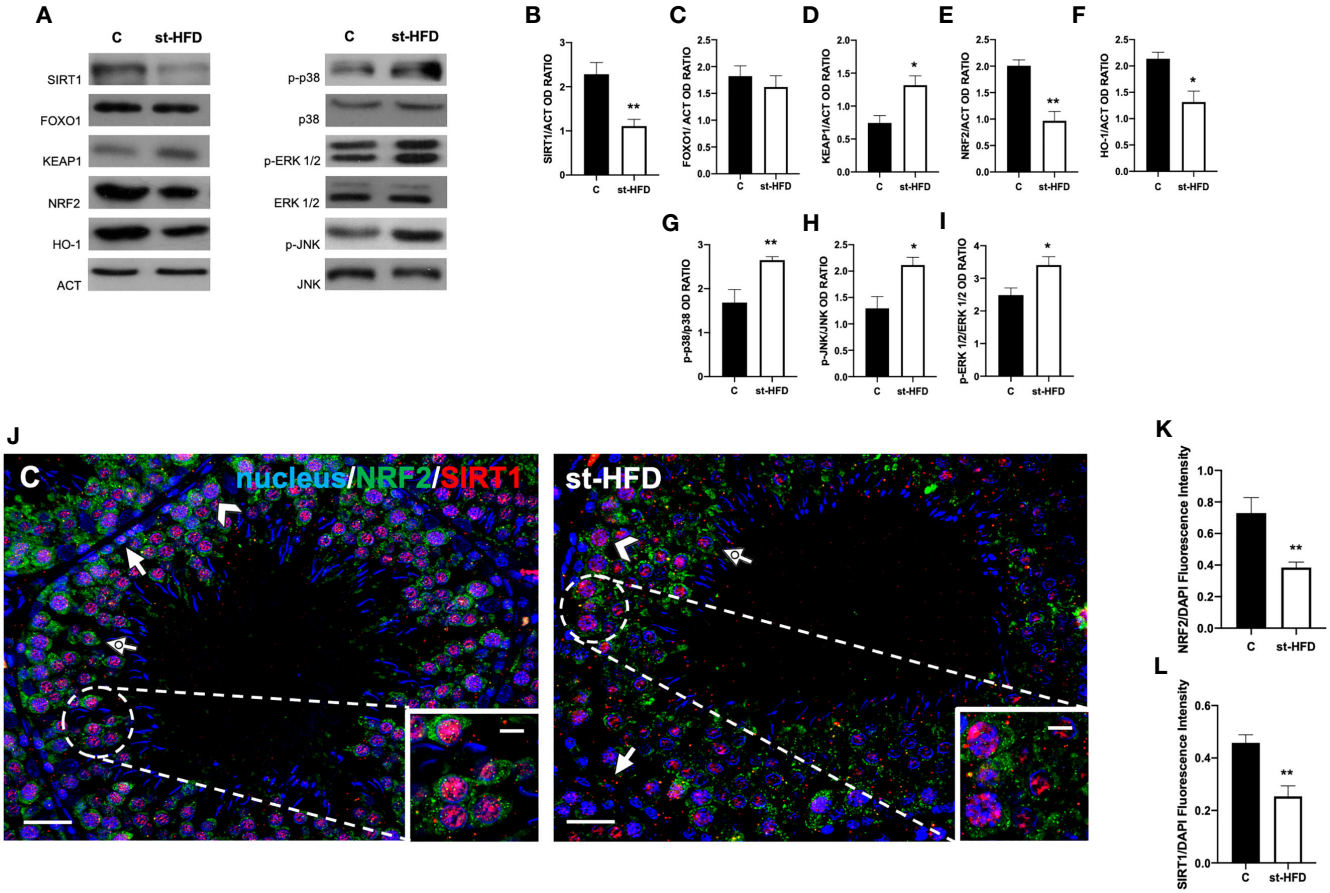
SIRT1/NRF2/MAPKs pathways analysis of control and st-HFD fed rat testis. **(A)** WB analysis of testicular SIRT1, FOXO1, KEAP1, NRF2, HO-1, p-p38, p38, p-ERK 1/2, ERK 1/2, p-JNK, and JNK. **(B–I)** Histograms showing SIRT1, FOXO1, KEAP1, NRF2, and HO-1 relative protein levels, and p-p38/p38, p-ERK 1/2/ERK 1/2, and p-JNK/JNK ratios. **(J)** Testicular NRF2 (green) and SIRT1 (red) immunolocalization. Slides were counterstained with DAPI-fluorescent nuclear staining (blue). The images were captured at x20 (scale bars= 20 µm) magnification and x40 (scale bars= 10 µm) for the insets. Arrows: SPG; Arrowheads: SPC Dotted arrows: SPT. **(K, L)** Histograms showing the quantification of NRF2 and SIRT1 fluorescence signal intensity, respectively. All the values are expressed as means ± SEM from 5 animals in each group. *p < 0.05; **p < 0.01.

In our previous paper, we assessed that st-HFD induced oxidative stress (42), thus herein we explored the underlying mechanisms, analyzing the SIRT1/NRF2/MAPKs pathways, that are notoriously involved in the cellular response to oxidative stress (65–69). Results showed that SIRT1 protein level decreased in st-HFD rat testis as compared to the control (p < 0.01; **Figures 9A, B**); conversely, no differences in FOXO1 levels were observed (**Figures 9A, C**).

The protein expression of KEAP1 increased in the st-HFD group (p < 0.05; **Figures 9A, D**), while those of NRF2 (p < 0.01; **Figures 9A, E**) and HO-1 (p < 0.05; **Figures 9A, F**) in the testis of st-HFD group were decreased compared with the control. Finally, the phosphorylation status of p38 (p< 0.01; **Figures 9A, G**), JNK (p < 0.05; **Figures 9A, H**), and ERK1/2 (p < 0.05; **Figures 9A, I**) was upregulated in the testis of st-HFD group as compared to the control.

To confirm these data, we performed double immunolabeling on SIRT1 and NRF2 in the two groups. In the control testis, SIRT1 possessed a nuclear localization, especially in SPG (arrows; **Figure 9J**), SPC (arrowheads; **Figure 9J**), and SPT (dotted arrow; **Figure 9J** and insets). On the contrary, although it was present in the same cells, NRF2 sub-localization was cytoplasmic (**Figure 9J**). In the testis of st-HFD rats, the intensity of both signals was weaker (p < 0.01; **Figures 9K, L**), particularly in the SPG nucleus for SIRT1 (arrows; **Figure 9J**) and in SPC cytoplasm for NRF2 (arrowheads; **Figure 9J**).

### 3.7 Effect of st-HFD on inflammation

**Figure 10:**
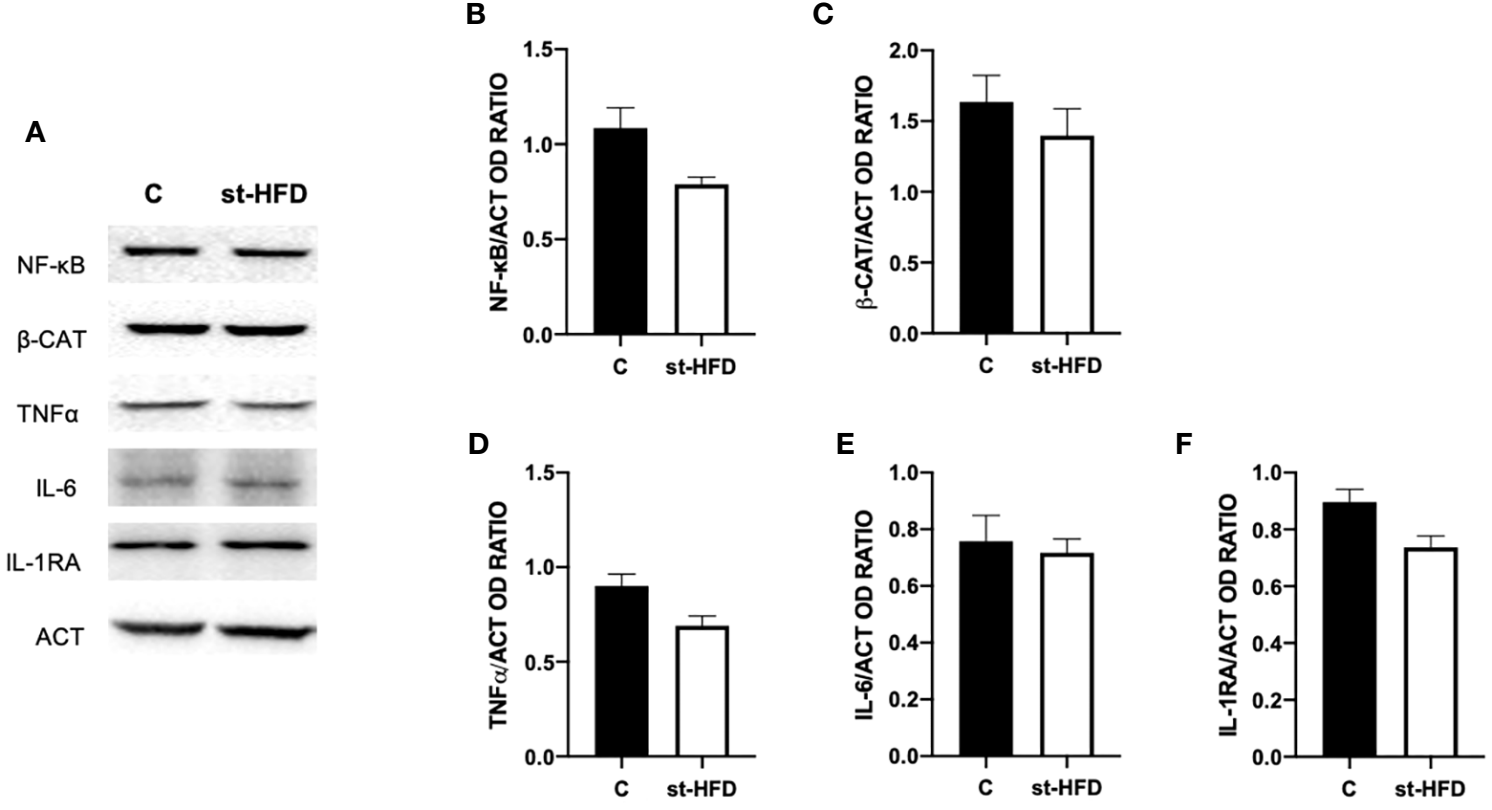
Inflammation markers analysis of control and st-HFD fed rat testis. **(A)** WB analysis of testicular NF-κB, β-Catenin, TNFα, IL-6, and IL-1RA. **(B–F)** Histograms showing NF-κB, β-Catenin, TNFα, IL-6, and IL-1RA relative protein levels. All the values are expressed as means ± SEM from 5 animals in each group.

To assess whether a st-HFD induced testicular inflammation, several markers, namely NF-κB (**Figures 10A, B**), β-catenin (β-CAT; **Figures 10A, C**), TNFα (**Figures 10A, D**), IL-6 (**Figures 10A, E**), and IL-1RA (**Figures 10A, F**) were used. Interestingly, there were no differences between st-HFD and control for any of the selected markers.

## 4 Discussion

Proper male and female reproductive activity are crucial for the health and survival of the species. This is accomplished by the production and differentiation of good quality gametes that, as for the male counterpart, are based on SPZ with the ability to cross the female genital tract, perform an accurate acrosome reaction, and contribute with an undamaged DNA for fertilization. Such events depend on an extremely intricate and specialized progression, which involves the proliferation (both mitotic and meiotic) of SPG into round SPT and their differentiation into SPZ, with also the contribution, for several aspects, of the somatic Sertoli and Leydig cells. Conversely, the decrease in sperm quality is a worldwide phenomenon, originating from a plethora of factors: genetic, environmental, and behavioral. Among the latter, dietary habits, with the spread of the so-called “Western diet” (characterized by being hypercaloric and nutritionally poor) is one of the most responsible, as a clear, multifunctional association between overweight/obesity and male sub- infertility has been extensively demonstrated (70–72). Indeed, many papers showed a positive correlation, in human and experimental rodent models fed with a long-term HFD, with increasing BMI and the worsening of several aspects related to fertility, as hormonal status (especially T level), sperm count, and motility, as well as the increased rate of oxidative stress and inflammation, increasing the risk of oligozoospermia and azoospermia (73, 74).

This work, with the use of a st-HFD rat model instead of the most usual mice/rats HFD-fed for a prolonged period, takes a different view, aimed to investigate the impact of overweight on testicular activity, since this condition represents the initial stage of the obesogenic process and may assess the status of affected people and direct them to a more correct diet (or other intervention strategies) in an attempt to mitigate its effects.

### 4.1 st-HFD alters testicular steroidogenesis and spermatogenesis

As expected, we found that the steroidogenesis was compromised in the testis of st-HFD rats. Herein, serum T levels were decreased significantly in the HFD group by about 28%, and our data agree with those by Migliaccio et al. (38), which evidenced a reduction in serum T levels and testicular androgen receptors in rats fed with a st-HFD (for 6 weeks). Nevertheless, the reduction in T levels was evidently less pronounced than that observed in rats fed with HFD for 12 (about 400%) (75), or 20 (about 180%) (35) weeks. On the contrary, we found no difference in ARO protein levels as compared to the control. Of note, a previous paper showed that 16 weeks of HFD induced an increase in serum E_2_ levels and testicular ARO expression (76) and, considering that this enzyme converts T into E_2_, and that decreased T/E_2_ ratio has been related to impaired spermatogenesis (56, 77–79), we highlighted that the disturbance of the hormonal milieu, induced by st-HFD, may be not so severe as that produced by a long term HFD. In addition, reduced T levels, together with the imbalance of oxidative status (42) are among the main causes of the negative impact induced by st-HFD on rat testis. Further, oxidative stress may also be one of the causes inducing LC apoptosis, exacerbating the reduced T bioavailability and, consequently, increasing the number of apoptotic GC. However, the apoptotic rate of testicular cells observed here was less pronounced as compared to that observed in the testis of HFD administered for a longer time (33–36, 80, 81), just confirming that an overweight-like condition provokes less detrimental effect as compared to that of obesity on testicular activity.

Our results showed that st-HFD impacts spermatogenic progression. While the histological organization was similar to controls, a reduced tubular diameter and epithelium thickness were observed. In addition, for the first time, we found significantly lower expression levels of SYCP3, an essential structural component of the synaptonemal complex, and PRM2, a protein associated with histone replacement in haploid cells during spermiogenesis (82). Vice versa, higher levels of PCNA, a nuclear antigen of cell proliferation, and p-H3, a histone protein crucial for chromatin condensation during mitosis/meiosis (83), were detected, together with a higher % of PCNA-positive SPG and I SPC. This last point is of interest, since our data are contrasting with that reported in other papers, in which a reduced number of PCNA-positive cells were observed in the seminiferous tubules of rats HFD-fed for 8 (36), 12 (84, 85), 18 (34), and 20 (35) weeks. Therefore, a st-HFD appeared to have a major negative effect on meiotic and post-meiotic events, rather than the previous ones. This data was partially supported by the fact that no differences in the frequency of stages characterizing rat seminiferous cycle were observed. This is in contrast with the paper by Komnions and colleagues (86), whose data demonstrated that a long-term HFD altered this value in mice; however, there were slight alterations in the phases of acrosome biogenesis. Further studies are required to clarify the underlying molecular aspects and the impact of a st-HFD on sperm parameters and physiology since proper acrosome formation is fundamental for successful fertilization (87).

### 4.2 st-HFD alters testicular mitochondrial dynamics via SIRT1 pathway

It is known that self-renewing and proliferating SPG use predominantly glycolysis, while in SPC and SPT, energy is prevalently produced through mitochondrial respiration, for this, fully functional mitochondria are required to complete a successful meiosis (43). Therefore, the altered progression of meiosis in st-HFD testis, as demonstrated by lower SYCP3 and PRM2 levels, could be the result of mitochondria damage, while the increased expression of PCNA and p-H3 in SPG and I SPC may be a compensatory response to the impaired maturation of GC. Bearing in mind the interesting data obtained by Migliaccio et al. (88), reporting that a st-HFD modifies mitochondrial fusion/fission processes in rat liver, we assessed whether the altered steroidogenesis/spermatogenesis in our animal model could also be induced by a consequence in mitochondrial dynamic changes. In particular, we analyzed several proteins involved in three pivotal mitochondrial processes: fusion (that promotes the maintenance of a homogeneous mitochondrial population that can tolerate higher levels of mitochondrial DNA mutations), fission (the division of a mitochondrion into two smaller mitochondria), and biogenesis (89). Our hypothesis on the involvement of mitochondrial damage in impaired spermatogenesis/steroidogenesis is confirmed by a decrease in MFN2 and OPA1 (fusion markers), DRP1 (fission marker), PGC-1α, NRF1, and TFAM (biogenesis marker) protein levels.

In this complex scenario, it should also be considered the multifaceted role played by SIRT1, a NAD^+^-dependent deacetylase, for several reasons (90). First, it has a well-recognized role in spermatogenesis, in particular to produce sex hormones by the hypothalamus-pituitary-testis axis (91) and for meiotic and post-meiotic progression (92). Second, SIRT1 is a ROS “sensor”, regulating, in oxidative stress conditions, the expression of several redox-related factors, such as FOXOs and NF-κB (90). Third, SIRT1 regulates mitochondrial function and energetic metabolism activating PGC-1α through deacetylation and mediating the induction of several components of the ROS detoxifying system (93). Fourth, testicular SIRT1 downregulation has previously been associated with the insurgence of an oxidative stress status (94) and in HFD-fed mice (53, 95). In view of these considerations, supporting earlier reports, we hypothesize that the effect of a st-HFD on impaired spermatogenesis may be also due to the downregulation of SIRT1 expression/activity and, consequently, of the downstream pathways, including those regulating mitochondrial dynamics.

### 4.3 st-HFD alters BTB integrity via NRF2/MAPKs pathways

BTB integrity is sensitive to stressful conditions, such as survival factor depletion and oxidative stress, as reported in several papers (96, 97). BTB is a distinctive structure of the testis, dividing the seminiferous epithelium into two compartments: the basal, where SPG and preleptotene SPC reside, and the apical one, which contains all the other cell types. It is composed of several cell junctions, located between adjacent SC, and particular cytoskeleton-based structures (the ES and the tubulobulbar complex), which connect SC to SPT. The BTB is an extremely dynamic structure, which, at stages IX–XI of the rat seminiferous epithelial cycle, is “disrupted” and then “reassembled” to permit the transit of preleptotene/leptotene SPC. This action is mediated by the interplay of various mechanisms that generally regulate fluctuation in the expression, localization, activation, and interactions of structural, scaffolding, and signaling proteins (61). Indeed, all the BTB components work harmoniously through continuous cycles of phosphorylation/de-phosphorylation, endocytosis of membrane proteins, and their recycling to guarantee the accurate moving of GC, and to preserve the immune-privileged microenvironment.

Herein, we confirmed that in the testis of st-HFD-fed rats, the protein levels of ZO-1, OCN, and CX43 were reduced (34). However, to our knowledge, this is the first report showing that a st-HFD affects testicular levels of N-CAD and VANGL2, proteins found at basal and apical ES, respectively, as well as the activation of Src and FAK.

In particular, FAK is a central kinase regulator of BTB dynamics, since its phosphorylation, by Src, at tyrosines 397 and 407, allows it to interact with many other components, including OCN, ZO-1, and Src itself. Once activated, FAK regulates the transit of GC through the seminiferous epithelium, especially maintaining the integrity of the apical ES and SPT adhesion during spermiogenesis until spermiation (98). Thus, as previously observed by other authors in HFD-fed mice for 10 (99), and 16 (100) weeks, we found that also a st-HFD can produce perturbations in BTB components, highlighting that its stability is fundamental for a correct spermatogenesis. However, as a limitation of this study, these are indirect data, and an *in vivo* BTB integrity assay would offer direct evidence, solidifying the claim.

### 4.4 st-HFD alters testicular activity via NRF2/MAPKs pathways

Emerging evidence demonstrated that the disturbance of BTB integrity may be due to ROS overproduction, by the downregulation of NRF2 (101) and activation of the MAPKs pathways (102, 103). Worth remembering, in physiological condition, NRF2 levels are maintained low via the repressive action of the protein KEAP1 while, in an oxidative stress environment, NRF2 is released by KEAP1, allowing its translocation into the nucleus, and activating the expression of antioxidant enzymes, including HO-1 and SOD. As for the MAPKs pathways, the increased activity of p38, JNK, and ERK 1/2 leads to OCN ubiquitination and degradation, as well as endocytosis of junction proteins, including N-CAD and CX43 (104–106).

In addition, it has also been reported that p38/JNK work together to activate the mitochondrial apoptotic pathway, via the stimulated expression of pro-apoptotic genes, such as cytochrome c and Caspase-3 (107, 108). Finally, apart from its well-known contribution to cell proliferation, numerous studies revealed that ERK 1/2 is also involved in apoptosis ROS-triggered (109–112). Consistently, our results showed that also a st-HFD induced the inhibition of the NRF2 pathway, as well as the phosphorylation, and thus the activation, of testicular p38, JNK, and ERK 1/2. These results were positively associated with the oxidative stress status and the enhanced apoptosis, while they were negatively correlated with the levels of structural proteins composing the BTB. The combined data suggest that BTB damage and apoptosis may be mediated by the inhibition of NRF2 and the activation of p38, JNK, and ERK 1/2 MAPK pathways, in st-HFD-fed rat testis, as already demonstrated in testicular tissues of type-1 diabetic or obese rodents (99, 113–116).

### 4.5 st-HFD does not induce testicular inflammation

Finally, for a broader picture of the effect of st-HFD on rat testis, the last analyzed parameter was the protein level of the pro-inflammatory markers NF-κB, β-CAT, TNFα, IL-6, and IL-1RA. However, no differences between st-HFD-treated rats and controls were found, and this point is particularly interesting, since one of the principal manifestations that are evidenced in obesity is the systemic inflammation, that produces altered testicular activity and sperm quality in men (114) and in rodents HFD-fed for a prolonged period (34, 84, 117–119). Thus, although a st-HFD can lead to dysfunction in testicular physiology, the lack of inflammation may be the sign of a less severe influence of overweight on fertility, suggesting that in overweight men there are still possibilities of intervention strategies (restricted diet, exercise, drugs, and others) that may effectively ameliorate testicular activity.

## 5 Conclusions

This study is one of the few to highlight the effects of a st-HFD on rat testicular activity. We demonstrated that disturbance in the hormonal milieu and the increased oxidative stress enhanced LC and GC apoptosis, reduced meiotic progression, and altered the integrity of BTB. These effects may be related to altered mitochondrial dynamics, and also to dysregulation of the SIRT1/NRF2/MAPKs pathways. However, we highlighted the absence of a claimed inflammation status, as well as the less % of TUNEL-positive cells, the increased % of PCNA-positive cells and no changes in the ARO protein level, as compared to literature papers in which a longer HFD was employed. The combined data led us to confirm that an overweight condition provoked less intense effects than obesity; however, as a limitation of this study, we lack a direct comparison with a long-term HFD, leading us to not completely exclude that these differences could be related to factors other than diet duration. In any case, this report encourages further studies not only to confirm this aspect but also on the development of different strategies to be used in preventing/mitigating the still not-so-severe effects of overweight on male fertility.

## Data availability statement

The original contributions presented in the study are included in the article/**Supplementary Material**. Further inquiries can be directed to the corresponding author.

## Ethics statement

The animal study was approved by Ethics Committee of the University of Campania “Luigi Vanvitelli” and the Italian Minister of Health (Permit Number: 704/2016-PR of the 15/07/2016; Project Number: 83700.1 of the 03/05/2015). The study was conducted in accordance with the local legislation and institutional requirements.

## Author contributions

SF: Formal Analysis, Investigation, Visualization, Writing – original draft. SM: Conceptualization, Supervision, Writing – review & editing. AS: Formal Analysis, Investigation, Visualization, Writing – original draft. RS: Methodology, Writing – original draft. GB: Conceptualization, Writing – review & editing. MV: Conceptualization, Formal Analysis, Funding acquisition, Project administration, Visualization, Writing – original draft.
