# Supplementary material for: A short-term high-fat diet alters rat testicular activity and blood-testis barrier integrity through the SIRT1/NRF2/MAPKs signaling pathways
Source: Front Endocrinol (Lausanne). 2023 Oct 27;14:1274035. doi: 10.3389/fendo.2023.1274035 (PMC10643185; doi:10.3389/fendo.2023.1274035)
Supplement: Supplementary file 1 [file Table_1.docx]

Supplementary Material

**Table 1.** List of all the used antibodies.

| **Antibody** | **Molecular weight (kDa)** | **WB Dilution** | **IF Dilution** | **Source** |
| --- | --- | --- | --- | --- |
| StAR | 32 | 1:700 | 1:100 | Elabscience Biotechnology, Wuhan, China  #E-AB-15419 |
| 3β-HSD | 42 | 1:700 | 1:100 | Elabscience Biotechnology, Wuhan, China  #E-AB-15112 |
| Aromatase | 48 | 1:1000 | - | Elabscience Biotechnology, Wuhan, China  #E-AB-68290 |
| PCNA | 36 | 1:1000 | 1:100 | Sigma-Aldrich, Milan, Italy  #98825 |
| phospho-Histone H3 | 17 | 1:1000 | - | Merck Millipore, Milan, Italy  #06–570 |
| Histone H3 | 17 | 1:1000 | - | Merck Millipore, Milan, Italy  #06–755 |
| SYCP3 | 30–33 | 1:250 | 1:50 | Santa Cruz Biotechnology, Santa Cruz, CA, USA  #sc-74569 |
| PRM2 | 13 | 1:500 | - | Proteintech, Manchester, UK  #14500-1-AP |
| PGC-1α | 130 | 1:1000 |  | Cell Signaling Technology, Danvers, MA, USA  #2178 |
| NRF1 | 68 | 1:1000 |  | Cell Signaling Technology, Danvers, MA, USA  #69432 |
| TFAM | 28 | 1:2000 |  | Abcam, Cambridge, UK  #ab131607 |
| MFN2 | 86 | 1:500 |  | Abcam, Cambridge, UK  ab56889 |
| OPA1 | 86-111 | 1:1000 |  | Abcam, Cambridge, UK  ab42364 |
| DRP1 | 78-82 | 1:1000 |  | Cell Signaling Technology, Danvers, MA, USA  #8570 |
| BAX | 21 | 1:750 | - | Elabscience Biotechnology, Wuhan, China  #E-AB-13814 |
| Bcl-2 | 22-26 | 1:750 | - | Elabscience Biotechnology, Wuhan, China  #E-AB-60012 |
| P53 | 43 | 1:500 | - | Elabscience Biotechnology, Wuhan, China  #E-AB-32469 |
| Caspase-3 | 17 | 1:700 | - | Elabscience Biotechnology, Wuhan, China  #E-AB-22115 |
| N-Cadherin | 125-135 | 1:1000 | 1:100 | Abcam, Cambridge, UK  #ab18203 |
| OCN | 65 | 1:1000 | 1:100 | Thermo Fisher Scientific, Waltham, MA, USA  #33-1500 |
| ZO-1 | 200 | 1:2000 | 1:100 | Thermo Fisher Scientific, Waltham, MA, USA  #40-2200 |
| CX43 | 43 | 1:1000 | 1:100 | Elabscience Biotechnology, Wuhan, China  #E-AB-70097 |
| VANGL2 | 60 | 1:500 | 1:100 | Sigma-Aldrich, Milan, Italy  #ABN2242 |
| p-Src | 60 | 1:1000 | - | Cell Signaling Technology, Danvers, MA, USA  #2101 |
| Src | 60 | 1:1000 | - | Cell Signaling Technology, Danvers, MA, USA  #2102 |
| p-FAK-Y397 | 125 | 1:500 | - | Thermo Fisher Scientific, Waltham, MA, USA  #44-625G |
| p-FAK-Y407 | 125 | 1:500 | - | Thermo Fisher Scientific, Waltham, MA, USA  #44-650G |
| FAK | 125 | 1:1000 | - | Thermo Fisher Scientific, Waltham, MA, USA  #39-6500 |
| SIRT1 | 120 | 1:3000 | 1:100 | Abcam, Cambridge, UK  #ab110304 |
| FOXO1 | 78 | 1:1000 | - | Elabscience Biotechnology, Wuhan, China  #E-AB-31466 |
| KEAP1 | 60 | 1:700 | - | ABclonal Science, Inc. Woburn, MA, USA  #A11258-20 |
| NRF2 | 68 | 1:2000 | 1:100 | GeneTex, Irvine, CA, USA  #GTX103322 |
| HO-1 | 33 | 1:1500 | - | GeneTex, Irvine, CA, USA  #GTX101147 |
| p-p38 | 41 | 1:500 | - | Sigma-Aldrich, Milan, Italy  #MABS64 |
| p38 | 41 | 1:1000 | - | ABclonal Science, Inc. Woburn, MA, USA  #A14401 |
| p-ERK1/2 | 42/44 | 1:1000 | - | Elabscience Biotechnology, Wuhan, China  #E-AB-20869 |
| ERK1/2 | 41/43 | 1:2000 | - | Elabscience Biotechnology, Wuhan, China  #E-AB-12397 |
| p-JNK | 46 | 1:500 | - | Santa Cruz Biotechnology, Santa Cruz, CA, USA  #sc-12882-R |
| JNK | 46 | 1:500 | - | Elabscience Biotechnology, Wuhan, China  #E-AB-60070 |
| NFkB p65 | 65 | 1:500 | - | Santa Cruz Biotechnology, Santa Cruz, CA, USA  #sc-8008 |
| β-Catenin | 92 | 1:1000 | - | Cell Signaling Technology, Danvers, MA, USA  #8480 |
| TNF α | 25 | 1:200 | - | Abcam, Cambridge, UK  #ab1793 |
| IL-6 | 25 | 1:1000 | - | Abcam, Cambridge, UK  #ab6672 |
| IL-1RA | 20 | 1:1000 | - | GeneTex, Irvine, CA, USA  #GTX106490 |
| β-Actin | 42 | 1:2000 | 1:100 | Elabscience Biotechnology, Wuhan, China  #E-AB-20031 |
| α-Tubulin | 52 | - | 1:100 | Elabscience Biotechnology, Wuhan, China  #E-AB-20036 |
| Goat anti-rabbit IgG HRP | - | 1:3000 | - | Vector Laboratories, Burlingame, CA, USA  #PI-1000 |
| Goat anti-mouse IgG HRP | - | 1:5000 | - | BioActs, Namdong-gu, Incheon, Korea  #RSA1122 |
| Goat anti-rabbit  Alexa Fluor 488 | - | - | 1:500 | Thermo Fisher Scientific, Waltham, Ma, USA  #A32731 |
| Goat anti-mouse  Alexa Fluor 647 | - | - | 1:500 | Thermo Fisher Scientific, Waltham, Ma, USA  #A21236 |
| PNA lectin   Alexa Fluor 568 | - | - | 1:50 | Thermo Fisher Scientific, Waltham, Ma, USA  #L32458 |

**
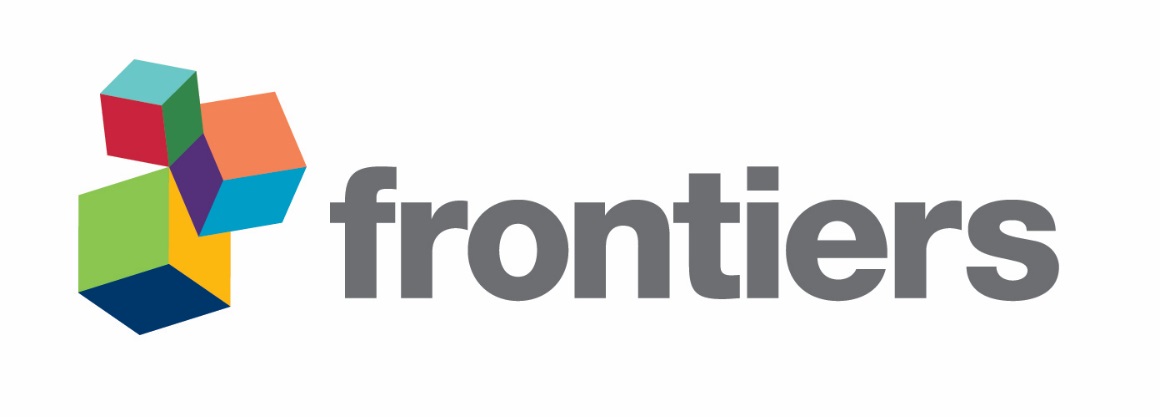
**
